# Association of childhood-to-adolescence body mass index trajectories with elevated blood pressure and elevated carotid intima-media thickness

**DOI:** 10.3389/fnut.2025.1562992

**Published:** 2025-10-29

**Authors:** Jintang Xie, Ziqi Liu, Min Zhao, Chuanwei Ma, Bo Xi

**Affiliations:** ^1^Department of Epidemiology/Department of Maternal, Child, Adolescent Health, School of Public Health, Qilu Hospital, Cheeloo College of Medicine, Shandong University, Jinan, China; ^2^Department of Nutrition and Food Hygiene, School of Public Health, Shandong University, Jinan, China; ^3^Department of Epidemiology and Health Statistics, School of Public Health, Guangdong Medical University, Dongguan, China

**Keywords:** body mass index, trajectory, blood pressure, carotid intima-media thickness, childhood, adolescence

## Abstract

**Background:**

Limited evidence exists on how early-life weight changes relate to cardiovascular damage in adolescents. We aimed to investigate the association between body mass index (BMI) trajectories from childhood to adolescence and elevated blood pressure (BP) and elevated carotid intima-media thickness (cIMT) in adolescents.

**Methods:**

This study included a total of 1,405 participants from the Huantai Children’s Cardiovascular Health Cohort who had at least two BMI measurements between 2017 and 2023. Group-based trajectory modeling was used to identify distinct BMI trajectories. Logistic regression models were used to analyze the association between these BMI trajectories and the development of elevated BP and elevated cIMT.

**Results:**

The BMI trajectory patterns of participants from childhood to adolescence were categorized into three groups: low-and-increasing (*n* = 473, 33.67%), medium-and-increasing (*n* = 533, 37.94%) and high-and-increasing (*n* = 399, 28.40%). Compared to the low-and-increasing group (systolic BP [SBP]: 110.16 mmHg, diastolic BP [DBP]: 60.59 mmHg, cIMT: 0.549 mm), the medium-and-increasing group had higher SBP (114.14 mmHg) and cIMT (0.567 mm), along with an increased risk of elevated BP (odds ratio [OR] 2.50, 95% confidence interval [CI] 1.43–4.39) and elevated cIMT (OR 3.17, 95% CI 1.50–6.74) (all *p* < 0.05). Similarly, the high-and-increasing group exhibited higher SBP (122.85 mmHg), DBP (62.83 mmHg), and cIMT (0.595 mm), as well as an increased risk of elevated BP (OR 10.73, 95% CI 6.23–18.48) and cIMT (OR 18.91, 95% CI 9.19–38.89) (all *p* < 0.05).

**Conclusion:**

Consistently elevated BMI from childhood to adolescence is closely associated with elevated BP and elevated cIMT during adolescence. Obesity prevention and screening in youth should be prioritized to reduce future cardiovascular disease risk.

## 1 Introduction

Over the past 30 years, the prevalence and burden of cardiovascular diseases (CVD) have significantly increased both in China and globally (1). From 1990 to 2021, CVD mortality and disability-adjusted life years in China increased by 68.4% (39.8–106.6) and 31.0% (9.1–59.5), respectively (2). In China, CVD has become the leading cause of death, with mortality rates rising from 234 per 100,000 in urban areas and 189 per 100,000 in rural areas in 2000, to 305 per 100,000 and 364 per 100,000, respectively, by 2021 (3). Strengthening the prevention and control of CVD is urgently needed.

It is well established that CVD often originates in early life (4, 5). Childhood obesity is associated with endothelial dysfunction, arterial stiffness, elevated carotid intima-media thickness (cIMT), and high left ventricular mass, collectively elevating the lifetime risk of CVD (6–8). Excessive body mass index (BMI) gain from childhood is significantly associated with the presence of CVD risk factors and an increased risk of CVD (9, 10). Additionally, BMI trajectories that are high or increasing from childhood to adulthood are associated with CVD risk factors in adulthood, including type 2 diabetes, hypertension, elevated cIMT, and dyslipidemia (11, 12).

Few studies have specifically explored the impact of BMI trajectories from childhood to adolescence on cardiovascular health. This study, therefore, aims to identify the patterns of BMI trajectories during this developmental period and to assess their association with early cardiovascular damage among Chinese adolescents, including elevated blood pressure (BP) (13) and elevated cIMT (14).

## 2 Methods

### 2.1 Study population and design

Data for this study were derived from the ongoing Huantai Children’s Cardiovascular Health Cohort, a prospective study (15). A convenience cluster sampling method was employed to select participants from an elementary school in Huantai County, Shandong Province, China. All children aged 6 to 11 years who voluntarily participated in the school were included in the study. The baseline survey was conducted in November 2017, with subsequent follow-ups every two years, resulting in a total of three follow-ups to date. The survey included questionnaires, physical examination and blood biochemistry tests. Written informed consent was obtained from both the participants and their guardians. The study was approved by the Ethics Committee of the School of Public Health, Shandong University (approval number: 20160308).

### 2.2 Procedures

Participants’ height and weight were measured using an ultrasonic height and weight scale (HGM 300, Universal Weight Electronics), with the average of two measurements used for analysis. BMI was calculated using the formula: BMI (kg/m^2^) = weight (kg) / [height (m)]^2^. BP was measured using validated electronic sphygmomanometers (OMRON HEM-7012 (16) at baseline, OMRON HBP-1300 (17) at follow-up), with three consecutive measurements taken and the average of the last two used for analysis to ensure reliability. Referring to the Chinese pediatric blood pressure reference standards established by Mi Jie et al. in 2107, we defined elevated BP as systolic or diastolic BP values at the last follow-up exceeding the 95^th^ percentile for gender, age, and height specific to Chinese children (18).

A specialized sonographer used a color Doppler ultrasound machine (Philips, CX30) to scan the right and left carotid arteries of the participants. The thicknesses of the left anterior wall, left posterior wall, right anterior wall, and right posterior wall of the carotid arteries were measured within a 5-mm range along the long-axis direction, approximately 1 cm proximal to the carotid bulb. The mean value of these measurements was taken as cIMT. Elevated cIMT was defined as cIMT values at the last follow-up exceeding the 90th percentile for gender and age within the cohort.

Information on participants’ sex, age, sleep duration, physical activity, and fruit and vegetable intake was obtained using a self-administered questionnaire. Sleep duration was defined as the average hours of sleep per night. Sufficient physical activity was defined as engaging in at least 1 h per day of vigorous, moderate physical activity, or walking (19). Sufficient fruit and vegetable intake was defined as consuming at least five servings per day (20). Fasting venous blood samples were obtained from participants, and levels of fasting blood glucose (FBG), triglycerides (TG), and total cholesterol (TC) were measured using an automatic analyzer (Beckman Coulter, AU480).

### 2.3 Statistical analyses

Statistical analyses were conducted using Stata (version 17.0, Sata Hard Drives) or R (version 4.3.0, R Core Team). Flowchart of inclusion/exclusion of participants is shown in Supplementary Figure 1. The Group-based Trajectory Model (GBTM) and the Stata “Traj” plugin were used to fit BMI trajectories from childhood to adolescence based on age- and sex-standardized BMI z-scores (21). We selected the three best-fitting quadratic trajectories based on expert knowledge and model selection criteria: (1) lower Bayesian information criterion (BIC); (2) average posterior probability for each group >0.7; (3) odds of correct classification >5; and (4) percentage of each group > 5% (22). The detailed statistical information of the models is shown in Supplementary Table 1.

Differences in characteristics between trajectory groups were compared using χ^2^ test for categorical variables and ANOVA for continuous variables, respectively. Adjusting for sex, age, sleep duration, physical activity, intake of fruits and vegetables, FBG, TG, and TC, differences in BP and cIMT values at last follow-up were compared across trajectory groups using ANCOVA, and odds ratios (ORs) for elevated BP and elevated cIMT were calculated using multivariable logistic regression model. Considering the effects of sex, physical activity, and fruit and vegetable intake on obesity and blood pressure (23–26), pre-specified subgroup analyses were conducted to assess potential differences by sex, physical activity, and intake of fruits and vegetables. Additionally, participants who were only followed up until 2019 were excluded for the sensitivity analysis.

## 3 Results

**Figure 1:**
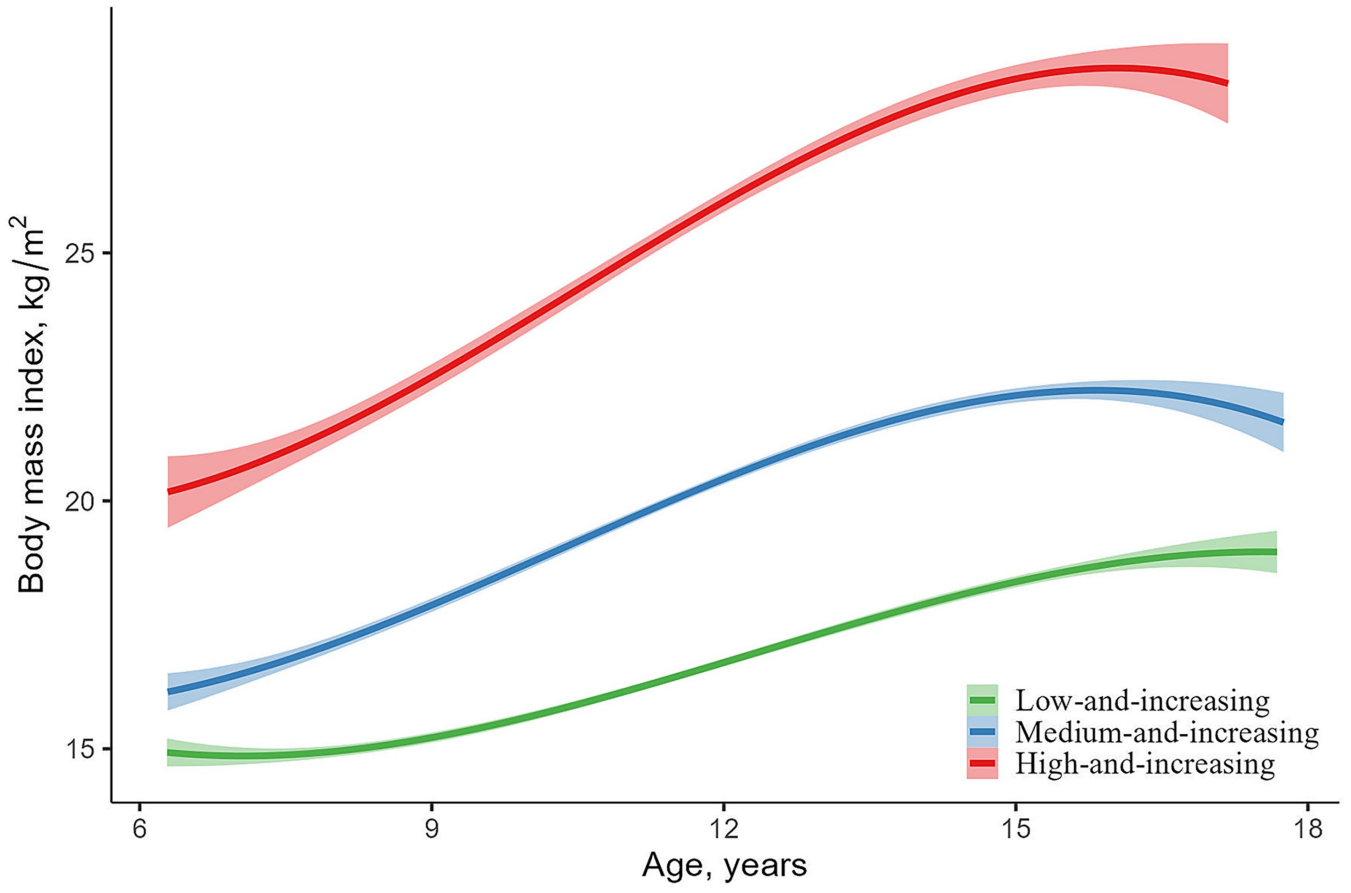
Trajectories of body mass index with age from childhood to adolescence.

After excluding participants not followed up, a total of 1,405 participants were included in the study. Three different trajectories were identified based on morphological characteristics: “low-and-increasing” (*n* = 473, 33.67%), “medium-and-increasing” (*n* = 533, 37.94%) and “high-and-increasing” (*n* = 399, 28.40%) (Figure 1). Although BMI increased in all three trajectories, the high-and-increasing group exhibited a greater rate and magnitude of increase. Table 1 presents the baseline characteristics of the trajectory groups and the BMI levels at each follow-up. Compared to the low-and-increasing group, the high-and-increasing group had a higher levels of height, weight, FBG, TG, and TC. Supplementary Tables 2, 3 present the differences in baseline characteristics between the included and excluded participants. Compared with the included participants, those who were excluded had higher baseline weight, total cholesterol, and BMI. No significant differences were observed for the other variables.

**Table 1:** Characteristics of participants by BMI trajectory groups^*^.

	Low-and-increasing (*n* = 473)	Medium-and-increasing (*n* = 533)	High-and-increasing (*n* = 399)	*P* value
Baseline				
Boys, *n* (%)	255 (53.91)	264 (49.53)	224 (56.14)	0.116
Age, years	8.74 (1.49)	8.82 (1.50)	8.83 (1.50)	0.569
Height, cm	132.75 (10.38)	135.65 (10.55)	138.57 (10.23)	<0.001
Weight, kg	27.16 (5.48)	32.85 (7.42)	43.17 (10.31)	<0.001
Sleep duration, hours/day	9.32 (0.49)	9.35 (0.49)	9.33 (0.46)	0.741
Sufficient physical activity, *n* (%)	167 (35.31)	213 (39.96)	141 (35.34)	0.217
Sufficient intake of fruits and vegetables, *n* (%)	91 (19.24)	95 (17.82)	66 (16.54)	0.584
FBG (mmol/l)	4.64 (0.52)	4.73 (0.56)	4.79 (0.53)	<0.001
TG (mmol/l)	0.66 (0.23)	0.71 (0.27)	0.92 (0.43)	<0.001
TC (mmol/l)	4.03 (0.71)	4.12 (0.83)	4.21 (0.81)	0.003
BMI, kg/m^2^				
BMI at baseline	15.24 (1.09)	17.60 (1.76)	22.15 (2.86)	<0.001
BMI at 1st follow-up	15.98 (1.35)	19.24 (1.91)	24.31 (2.68)	<0.001
BMI at 2nd follow-up	17.13 (1.54)	20.86 (1.86)	26.80 (3.11)	<0.001
BMI at 3rd follow-up	18.13 (1.70)	22.02 (2.16)	28.23 (3.80)	<0.001

^*^Based on age- and sex-standardized BMI z-score; Data are presented as mean (standard deviation) for continuous variables and *n* (%) for categorical variables. BMI, body mass index; FBG, fasting blood glucose; TC, total cholesterol; TG, triglyceride.

**Figure 2:**
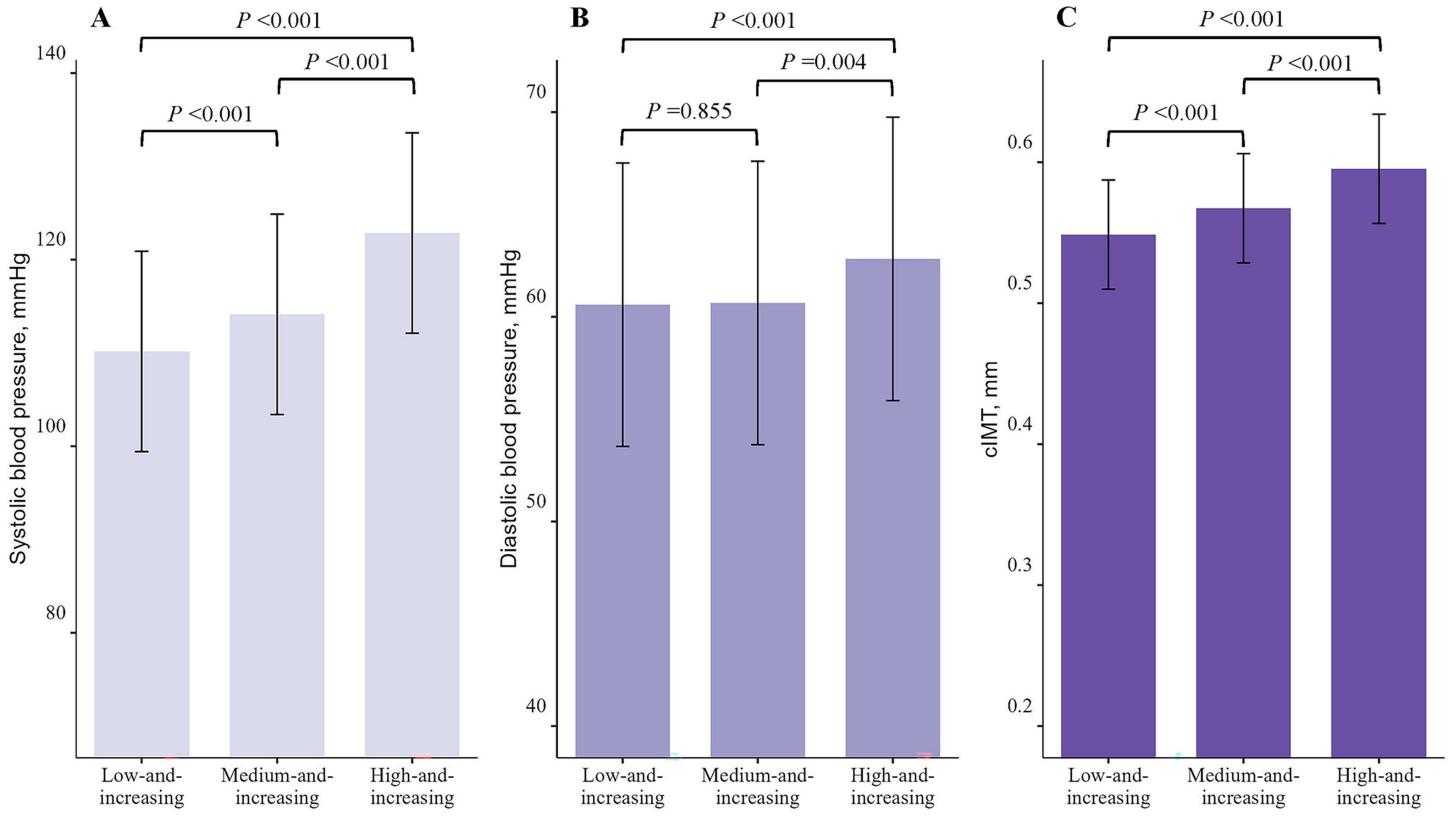
Levels of systolic blood pressure, diastolic blood pressure, and carotid intima-media thickness in body mass index trajectory groups at last follow-up. **(A)** systolic blood pressure; **(B)** diastolic blood pressure; **(C)** carotid intima-media thickness.

Compared to the low-and-increasing group (systolic BP [SBP]: 110.16 mmHg, diastolic BP [DBP]: 60.59 mmHg, cIMT: 0.549 mm), the medium-and-increasing group exhibited higher SBP (114.14 mmHg) and cIMT (0.567 mm) at the last follow-up. The high-and-increasing group showed even higher levels of SBP (122.85 mmHg), DBP (62.83 mmHg), and cIMT (0.595 mm) (all *p* < 0.05, Figure 2).

After adjusting for potential covariates, the risk of elevated BP and elevated cIMT was 2.50 times greater (95% confidence interval [CI]1.43–4.39) and 3.17 times greater (95% CI 1.50–6.74), respectively, in the medium-and-increasing group, and 10.73 times greater (95% CI 6.23–18.48) and 18.91 times greater (95% CI 9.19–38.89), respectively, in the high-and-increasing group compared to the low-and-increasing group (Table 2). Subgroup analyses and sensitivity analyses excluding participants (data not shown) who were only followed up until 2019 yielded similar results (Supplementary Tables 4–7).

**Table 2:** Association of BMI trajectory groups with elevated BP and elevated cIMT.

	*n* (%)	Model 1		Model 2	
		OR (95% CI)	*P* value	OR (95% CI)	*P* value
Elevated BP					
Low-and-increasing	19 (4.35)	1.00		1.00	
Medium-and-increasing	44 (9.78)	2.47 (1.41, 4.31)	0.002	2.50 (1.43, 4.39)	0.001
High-and-increasing	96 (32.32)	10.55 (6.25, 17.83)	<0.001	10.73 (6.23, 18.48)	<0.001
Elevated cIMT					
Low-and-increasing	9 (1.95)	1.00		1.00	
Medium-and-increasing	32 (6.21)	3.27 (1.54, 6.92)	0.002	3.17 (1.50, 6.74)	0.003
High-and-increasing	85 (30.47)	22.12 (10.90, 44.90)	<0.001	18.91 (9.19, 38.89)	<0.001

BMI, body mass index; BP, blood pressure; CI, confidence interval; cIMT, carotid intima-media thickness; OR, odds ratio. Model 1 adjusted for sex and age at baseline; Model 2 adjusted for sex, age, sleep duration, physical activity, intake of fruits and vegetables, fasting blood glucose, triglyceride and total cholesterol at baseline.

## 4 Discussion

Our study observed a general upward trend in BMI from childhood to adolescence, with particularly pronounced increases in the medium-and-increasing and high-and-increasing groups compared to the low-and-increasing group. Moreover, compared to the low-and-increasing group, both the medium-and-increasing group and high-and-increasing groups exhibited higher levels of SBP, DBP, and cIMT, and were at a significantly increased risk of developing elevated BP and elevated cIMT.

Obesity and rapid weight gain during childhood and adolescence are associated with an increased risk of CVD in adulthood (27). There is heterogeneity in BMI trajectory patterns with age, and different trajectory subgroups may be associated with distinct pathogenesis, health hazards, and intervention strategies (28). In this study, using GBTM, we identified three BMI trajectory patterns from childhood to adolescence: 34.9% of participants showed a low and steadily increasing trend, labeled as the “low-and-increasing” group; 36.2% had a moderate initial BMI with a slightly faster growth rate, labeled as the “medium-and-increasing” group; and 28.9% had a high initial BMI that remained elevated over time, labeled as the “high-and-increasing” group. The high-and-increasing group had a significantly higher risk of developing elevated BP and elevated cIMT during adolescence.

Our findings are comparable to previous studies that also identified three BMI trajectory groups. For instance, the Hanzhong Adolescent Hypertension Study (*n* = 1,824, 1987–2013) reported three trajectories from childhood to adulthood: low-increasing (26.9%), moderate-increasing (58.8%), and high-increasing (14.4%) (22). Participants in the Hanzhong cohort with a moderate-increasing (OR = 1.49, 95% CI 1.00–2.23) or high-increasing (OR = 2.45, 95% CI 1.22–4.91) trajectory had a significantly higher risk of metabolic syndrome in middle age compared to those with a low-increasing trajectory (22). Similarly, the Cardiovascular Risk in Young Finns Study (*n* = 2,631) demonstrated that persistently high BMI trajectories from childhood to adulthood were associated with markedly increased risks of hypertension (RR = 2.98, 95% CI 1.51–5.02) and elevated cIMT (RR = 3.14, 95% CI 2.21–4.12) in adulthood (11). Although the overall trajectory patterns are consistent, the distribution of participants across groups differed. In the Hanzhong cohort, 13.8% of participants were in the high-increasing trajectory, whereas our study observed a relatively larger proportion in the high-and-increasing group (28.9%). These discrepancies may reflect secular trends in obesity prevalence, as the burden of childhood overweight and obesity in China has risen substantially since the 1990s (29). Differences in cohort characteristics, such as birth years, socioeconomic context, diet, and physical activity, as well as the different follow-up windows (our study: childhood to adolescence; Hanzhong and Young Finns studies: childhood to adulthood), may also contribute (30). Collectively, the evidence from our study and previous cohorts consistently highlights that elevated BMI trajectories from early life substantially increase the risk of adverse cardiometabolic outcomes, underscoring the importance of weight management beginning in childhood.

Obesity increases total blood volume, cardiac output, and stroke volume, thereby placing an additional burden on the vascular and cardiac systems (31). Moreover, obese children exhibit elevated levels of pro-inflammatory cytokines and worsened endothelial dysfunction compared to normal-weight peers, which may contribute to an increased risk of CVD (32). Evidence suggests that the cardiovascular consequences of obesity are cumulative, and the duration of being overweight or obese is a strong predictor of CVD (33, 34). A study based on the Framingham Cohort Study showed that the duration of obesity is directly associated with mortality risk (35). For every additional 2 years of obesity, the hazard ratios for all-cause and CVD mortality were 1.06 (95% CI 1.05–1.07) and 1.07 (95% CI 1.05–1.08), respectively (35). These findings highlight the significant and lasting impact of childhood obesity on cardiovascular health, underscoring the importance of early intervention and prevention strategies.

This prospective cohort study explores the association between BMI trajectories from childhood to adolescence and the development of elevated BP and elevated cIMT during adolescence. The identification of distinct BMI trajectory patterns and their associations with early markers of cardiovascular damage highlights the urgent need for early-life obesity prevention and intervention strategies. Targeted public health initiatives that promote healthy growth trajectories, especially in children with high or rapidly increasing BMI, could help mitigate the future burden of cardiovascular disease. School- and community-based programs focusing on balanced nutrition, regular physical activity, and health education may be particularly effective in high-risk populations (36). Future research could explore the effectiveness of early, targeted interventions, such as lifestyle modification programs, for children identified as having high-risk BMI trajectories. In addition, integrating routine BMI trajectory monitoring into school or community health programs could facilitate early detection and personalized prevention strategies. Long-term follow-up studies are also warranted to evaluate the sustained impact of these interventions on cardiovascular health outcomes in adulthood.

However, there are several limitations to consider. First, the follow-up period was relatively short, with some participants being followed up only once or twice. Fortunately, sensitivity analysis excluding participants who were followed up only during the first visit yielded similar results. Second, all participants were from the same primary school, so our findings should be generalized with caution. Third, data on sleep duration, physical activity, and fruit and vegetable intake were self-reported by participants, which may have introduced reporting bias. Fourth, due to data limitations, the influence of some unmeasured confounders, such as dietary patterns (37) and mental health (38), could not be excluded.

## 5 Conclusion

In conclusion, the BMI trajectory from childhood to adolescence generally shows an upward trend, and those with persistently high levels of BMI are closely associated with elevated BP and elevated cIMT during adolescence. Therefore, it is essential to implement longitudinal and continuous screening and prevention measures for children and adolescents to reduce the risk of future CVD.

## Data Availability

The raw data supporting the conclusions of this article will be made available by the authors, without undue reservation.
