# Supplementary material for: Association of childhood-to-adolescence body mass index trajectories with elevated blood pressure and elevated carotid intima-media thickness
Source: Front Nutr. 2025 Oct 29;12:1562992. doi: 10.3389/fnut.2025.1562992 (PMC12604995; doi:10.3389/fnut.2025.1562992)
Supplement: Supplementary file 1 [file Supplementary_file_1.docx]

Supplementary Material

# Supplementary Figures and Tables

## Supplementary Figures





**Supplementary Figure 1.** Analytical flowchart of the study.

## Supplementary Tables

**Supplementary Table 1.** General statistical information of group-based trajectory modeling in this study^*^

| Group numbers | Shape of trajectory | BIC (n=5191) | BIC (n=1405) | Average posterior probabilities | Odds of correct classification | Group proportion, % |
| --- | --- | --- | --- | --- | --- | --- |
| 1 | linear | -7344.24 | -7342.28 | - | - | - |
| 1 | quadratic | -7348.49 | -7345.88 | - | - | - |
| 1 | cubic | -7352.69 | -7349.43 | - | - | - |
| 2 | linear | -5345.61 | -5341.04 | 0.98/0.97 | 25.5/54.88 | 62.14/37.86 |
| 2 | quadratic | -5351.97 | -5346.09 | 0.98/0/97 | 24.11/58.65 | 62.14/37.86 |
| 2 | cubic | -5360.27 | -5353.08 | 0.98/0.97 | 23.95/58.78 | 62.14/37.86 |
| 3 | linear | -4535.74 | -4528.56 | 0.96/0.93/0.97 | 46.68/22.26/82.74 | 34.16/37.58/28.26 |
| **3** | **quadratic** | **-4527.99** | **-4518.84** | **0.96/0.93/0.97** | **44.65/23.10/80.95** | **33.67/37.94/28.40** |
| 3 | cubic | -4539.91 | -4528.81 | 0.96/0.93/0.97 | 45.55/22.64/83.73 | 33.67/38.01/28.33 |

^*^Based on age- and sex-standardized BMI z-score;

BIC, Bayesian Information Criterion

**Supplementary Table 2.** Baseline characteristics of participants included and excluded in performing analysis related to elevated BP

| Characteristics | Included (n=1184) | Excluded (n=332) | *P* value |
| --- | --- | --- | --- |
| Boys, n (%) | 628 (53.0) | 181 (54.5) | 0.633 |
| Age, years | 8.8 (1.5) | 9.3 (1.5) | 0.989 |
| Height, cm | 135.2 (10.8) | 139.5 (10.0) | 0.093 |
| Weight, kg | 33.1 (9.8) | 39.1 (11.2) | 0.002 |
| Sleep duration, hours/day | 9.3 (0.5) | 9.3 (0.5) | 0.148 |
| Sufficient physical activity, n (%) | 437 (36.9) | 136 (41.0) | 0.191 |
| Sufficient intake of fruits and vegetables, n (%) | 219 (18.5) | 62 (18.7) | 0.941 |
| FBG (mmol/l) | 4.7 (0.6) | 4.8 (0.6) | 0.661 |
| TG (mmol/l) | 0.7 (0.3) | 0.9 (0.4) | <0.001 |
| TC (mmol/l) | 4.1 (0.8) | 4.1 (0.8) | 0.810 |
| BMI, kg/m^2^ | 17.8 (3.2) | 19.8 (3.9) | <0.001 |

Data are mean (standard deviation) for continuous variables and n (%) for categorical variables.

BMI, body mass index; BP, blood pressure; FBG, fasting blood glucose; TC, total cholesterol; TG, triglyceride.

**Supplementary Table 3.** Baseline characteristics of participants included and excluded in performing analysis related to elevated cIMT

| Characteristics | Included (n=1255) | Excluded (n=261) | *P* value |
| --- | --- | --- | --- |
| Boys, n (%) | 664 (52.9) | 145 (55.6) | 0.435 |
| Age, years | 8.8 (1.5) | 9.4 (1.5) | 0.542 |
| Height, cm | 134.9 (10.6) | 142.0 (9.5) | 0.027 |
| Weight, kg | 32.6 (9.1) | 43.0 (12.0) | <0.001 |
| Sleep duration, hours/day | 9.3 (0.5) | 9.3 (0.5) | 0.967 |
| Sufficient physical activity, n (%) | 477 (38.0) | 96 (36.1) | 0.710 |
| Sufficient intake of fruits and vegetables, n (%) | 224 (07.9) | 57 (21.8) | 0.131 |
| FBG (mmol/l) | 4.7 (0.6) | 4.9 (0.6) | 0.907 |
| TG (mmol/l) | 0.7 (0.3) | 1.0 (0.5) | <0.001 |
| TC (mmol/l) | 4.1 (0.8) | 4.2 (0.9) | 0.135 |
| BMI, kg/m^2^ | 17.6 (2.9) | 21.1 (4.2) | <0.001 |

Data are mean (standard deviation) for continuous variables and n (%) for categorical variables.

BMI, body mass index; cIMT, carotidintima-media thickness; FBG, fasting blood glucose; TC, total cholesterol; TG, triglyceride.

**Supplementary Table 4.** Association of BMI trajectory groups with elevated BP and elevated cIMT by sex

|  | n (%) | Model 1 | |  | Model 2 | |
| --- | --- | --- | --- | --- | --- | --- |
|  |  | OR (95% CI) | *P* value |  | OR (95% CI) | *P* value |
| **Boys** |  |  |  |  |  |  |
| Elevated BP |  |  |  |  |  |  |
| Low-and-increasing | 17 (7.20) | 1.00 |  |  | 1.00 |  |
| Medium-and-increasing | 32 (14.22) | 2.14 (1.15, 3.98) | 0.016 |  | 2.19 (1.17, 4.11) | 0.014 |
| High-and-increasing | 60 (35.93) | 7.07 (3.93, 12.71) | <0.001 |  | 7.42 (4.02, 13.71) | <0.001 |
| Elevated cIMT |  |  |  |  |  |  |
| Low-and-increasing | 4 (1.59) | 1.00 |  |  | 1.00 |  |
| Medium-and-increasing | 16 (6.23) | 4.11 (1.35, 12.46) | 0.013 |  | 3.94 (1.29, 12.01) | 0.016 |
| High-and-increasing | 41 (26.45) | 22.17 (7.76, 63.41) | <0.001 |  | 18.53 (6.36, 54.02) | <0.001 |
| **Girls** |  |  |  |  |  |  |
| Elevated BP |  |  |  |  |  |  |
| Low-and-increasing | 2 (1.00) | 1.00 |  |  | 1.00 |  |
| Medium-and-increasing | 12 (5.33) | 5.46 (1.21, 24.72) | 0.027 |  | 5.49 (1.21, 24.96) | 0.010 |
| High-and-increasing | 36 (27.69) | 38.57 (9.08, 163.89) | <0.001 |  | 37.74 (8.67, 164.16) | <0.001 |
| Elevated cIMT |  |  |  |  |  |  |
| Low-and-increasing | 5 (2.39) | 1.00 |  |  | 1.00 |  |
| Medium-and-increasing | 16 (6.20) | 2.67 (0.96, 7.43) | 0.059 |  | 2.66 (0.95, 7.43) | 0.062 |
| High-and-increasing | 44 (35.48) | 22.52 (8.61, 58.87) | <0.001 |  | 20.45 (7.65, 54.66) | <0.001 |

BMI, body mass index; BP, blood pressure; CI, confidence interval; cIMT, carotid intima-media thickness; OR, odds ratio.

Model 1 adjusted for sex and age at baseline; Model 2 adjusted for sex, age, sleep duration, physical activity, intake of fruits and vegetables, fasting blood glucose, triglyceride and total cholesterol at baseline.

**Supplementary Table 5.** Association of BMI trajectory groups with elevated BP and elevated cIMT by physical activity

|  | n (%) | Model 1 | |  | Model 2 | |
| --- | --- | --- | --- | --- | --- | --- |
|  |  | OR (95% CI) | *P* value |  | OR (95% CI) | *P* value |
| **Insufficient physical activity** |  |  |  |  |  |  |
| Elevated BP |  |  |  |  |  |  |
| Low-and-increasing | 14 (4.98) | 1.00 |  |  | 1.00 |  |
| Medium-and-increasing | 25 (9.03) | 1.89 (0.96, 3.73) | 0.067 |  | 1.88 (0.95, 3.73) | 0.071 |
| High-and-increasing | 57 (30.16) | 8.04 (4.30, 15.06) | <0.001 |  | 7.96 (4.16, 15.25) | <0.001 |
| Elevated cIMT |  |  |  |  |  |  |
| Low-and-increasing | 7 (2.36) | 1.00 |  |  | 1.00 |  |
| Medium-and-increasing | 17 (5.48) | 2.38 (0.97, 5.84) | 0.057 |  | 2.38 (0.97, 5.86) | 0.059 |
| High-and-increasing | 53 (30.99) | 18.72 (8.26, 42.42) | <0.001 |  | 16.48 (7.16, 37.94) | <0.001 |
| **Sufficient physical activity** |  |  |  |  |  |  |
| Elevated BP |  |  |  |  |  |  |
| Low-and-increasing | 5 (3.21) | 1.00 |  |  | 1.00 |  |
| Medium-and-increasing | 19 (10.98) | 4.24 (1.53, 11.75) | 0.006 |  | 4.80 (1.68, 13.70) | 0.003 |
| High-and-increasing | 39 (36.11) | 18.58 (6.94, 49.76) | <0.001 |  | 23.58 (8.20, 67.79) | <0.001 |
| Elevated cIMT |  |  |  |  |  |  |
| Low-and-increasing | 2 (1.22) | 1.00 |  |  | 1.00 |  |
| Medium-and-increasing | 15 (7.32) | 6.05 (1.36, 26.91) | 0.018 |  | 5.96 (1.33, 26.58) | 0.019 |
| High-and-increasing | 32 (29.63) | 33.62 (7.84, 144.12) | <0.001 |  | 28.60 (6.50, 125.86) | <0.001 |

BMI, body mass index; BP, blood pressure; CI, confidence interval; cIMT, carotid intima-media thickness; OR, odds ratio.

Model 1 adjusted for sex and age at baseline; Model 2 adjusted for sex, age, sleep duration, physical activity, intake of fruits and vegetables, fasting blood glucose, triglyceride and total cholesterol at baseline.

**Supplementary Table 6.** Association of BMI trajectory groups with elevated BP and elevated cIMT by intake of fruits and vegetables

|  | n (%) | Model 1 | |  | Model 2 | |
| --- | --- | --- | --- | --- | --- | --- |
|  |  | OR (95% CI) | *P* value |  | OR (95% CI) | *P* value |
| **Insufficient intake of fruits and vegetables** | | | | | | |
| Elevated BP |  |  |  |  |  |  |
| Low-and-increasing | 15 (4.25) | 1.00 |  |  | 1.00 |  |
| Medium-and-increasing | 37 (10.00) | 2.52 (1.35, 4.69) | 0.004 |  | 2.57 (1.37, 4.80) | 0.003 |
| High-and-increasing | 79 (32.64) | 10.86 (6.03, 19.54) | <0.001 |  | 10.99 (5.97, 20.23) | <0.001 |
| Elevated cIMT |  |  |  |  |  |  |
| Low-and-increasing | 4 (4.76) | 1.00 |  |  | 1.00 |  |
| Medium-and-increasing | 7 (8.75) | 5.21 (1.99, 13.65) | <0.001 |  | 5.01 (1.91, 13.15) | 0.001 |
| High-and-increasing | 17 (30.91) | 32.13 (12.73, 81.08) | <0.001 |  | 26.46 (10.37, 67.53) | <0.001 |
| **Sufficient intake of fruits and vegetables** | | | | | | |
| Elevated BP |  |  |  |  |  |  |
| Low-and-increasing | 5 (1.33) | 1.00 |  |  | 1.00 |  |
| Medium-and-increasing | 28 (6.64) | 2.29 (0.63, 8.30) | 0.208 |  | 2.05 (0.54, 7.76) | 0.289 |
| High-and-increasing | 71 (30.34) | 9.17 (2.86, 29.40) | <0.001 |  | 9.38 (2.72, 32.33) | <0.001 |
| Elevated cIMT |  |  |  |  |  |  |
| Low-and-increasing | 4 (4.65) | 1.00 |  |  | 1.00 |  |
| Medium-and-increasing | 4 (4.30) | 0.79 (0.19, 3.31) | 0.750 |  | 0.83 (0.19, 3.52) | 0.796 |
| High-and-increasing | 14 (31.11) | 10.69 (3.16, 36.16) | <0.001 |  | 11.40 (3.05, 42.64) | <0.001 |

BMI, body mass index; BP, blood pressure; CI, confidence interval; cIMT, carotid intima-media thickness; OR, odds ratio.

Model 1 adjusted for sex and age at baseline; Model 2 adjusted for sex, age, sleep duration, physical activity, intake of fruits and vegetables, fasting blood glucose, triglyceride and total cholesterol at baseline.

**Supplementary Table 7.** Sensitivity analysis of association of BMI trajectory groups with elevated BP and elevated cIMT by intake of fruits and vegetables

|  | n (%) | Model 1 | |  | Model 2 | |
| --- | --- | --- | --- | --- | --- | --- |
|  |  | OR (95% CI) | *P* value |  | OR (95% CI) | *P* value |
| **Elevated BP** |  |  |  |  |  |  |
| Low-and-increasing | 19 (4.49) | 1.00 |  |  | 1.00 |  |
| Medium-and-increasing | 44 (10.11) | 2.47 (1.41, 4.32) | 0.002 |  | 2.53 (1.44, 4.45) | 0.001 |
| High-and-increasing | 92 (32.39) | 10.29 (6.07, 17.43) | <0.001 |  | 10.68 (6.17, 18.47) | <0.001 |
| **Elevated cIMT** |  |  |  |  |  |  |
| Low-and-increasing | 9 (2.02) | 1.00 |  |  | 1.00 |  |
| Medium-and-increasing | 31 (6.21) | 3.16 (1.49, 6.73) | 0.003 |  | 3.08 (1.45, 6.55) | 0.004 |
| High-and-increasing | 82 (30.83) | 21.71 (10.67, 44.17) | <0.001 |  | 18.80 (9.12, 38.75) | <0.001 |

BMI, body mass index; BP, blood pressure; CI, confidence interval; cIMT, carotid intima-media thickness; OR, odds ratio.

Model 1 adjusted for sex and age at baseline; Model 2 adjusted for sex, age, sleep duration, physical activity, intake of fruits and vegetables, fasting blood glucose, triglyceride and total cholesterol at baseline.
